# Risk Factors Associated with Under-Five Stunting, Wasting, and Underweight Based on Ethiopian Demographic Health Survey Datasets in Tigray Region, Ethiopia

**DOI:** 10.1155/2019/6967170

**Published:** 2019-12-20

**Authors:** Berhanu Teshome Woldeamanuel, Tigist Tigabie Tesfaye

**Affiliations:** ^1^Department of Statistics, Salale University, P.O. Box: 254, Fiche, Ethiopia; ^2^Department of Statistics, Mekelle University, P.O. Box: 231, Mek'ele, Ethiopia

## Abstract

**Background:**

Stunting, wasting, and underweight among children are major problems in most regions of Ethiopia, including the Tigray region. The main objective of this study was to assess the risk factors associated with stunting, wasting, and underweight of children in the Tigray region.

**Methods:**

The information collected from 1077 children born 5 years before the survey was considered in the analysis. Multivariable binary logistic regression analysis was fitted to identify significant risk factors associated with stunting, wasting, and underweight.

**Results:**

Male children and rural born were having a higher burden of both severe and moderate stunting, wasting, and underweight than females and urban born. Among male children, 27.6%, 4.10%, and 14.2% of them were stunted, wasted, and underweight, respectively. Protected drinking water (odds ratio (OR) = 0.68; 95% confidence interval (CI): (0.50, 0.92)) was associated with stunting. Maternal age at birth less than 20 years (OR = 0.66; 95% CI: (0.45, 0.97)) and being male (OR = 2.04; 95% CI: (1.13, 3.68)) were associated with high risk of underweight. No antenatal care follow-up (OR = 2.20; 95% CI: (1.04, 4.64)) was associated with wasting, while the poor wealth index, diarrhea, low weight at birth (<2.5 kg), lower age of a child, and 3 or more under-five children in a household were significantly associated with stunting, wasting, and underweight.

**Conclusions:**

Being born in rural, being male, unprotected drinking water, smaller weight at birth, no antenatal follow-ups, diarrhea, and poor household wealth were factors associated with increased stunting, wasting, and underweight. Thus, interventions that focus on utilization of antenatal care services, improving household wealth, and improving access to protected drinking water were required by policymakers to decrease stunting, wasting, and underweight more rapidly.

## 1. Introduction

Globally, about 151 million children under 5 years of age suffer from chronic malnutrition and 67 million under-five were wasted in 2017 [1]. Among these malnourished under-five, 55% of all stunted children under 5 years of age have been from Asia and 39% were living in Africa, while 69% of all wasted children under 5 years of age resided in Asia and 27% of wasted under-five lived in Africa. Moreover, UNICEF, WHO, and World Bank Group Joint Child Malnutrition estimate in 2018 that about 35.60% of under-five in east Africa were stunted [2].

Malnutrition among under-five declined from 32.6% to 22.2% between 2000 and 2017 worldwide [3]. In 2018, UNICEF reported that about 3.1 million children died of undernutrition and malnutrition contributes to more than half of global child deaths [4]. Though there is a global decline in the malnutrition rates of under-5, the risk of malnutrition remains high and it is the major cause of under-five morbidities and mortalities of African especially sub-Saharan Africa [5]. Burundi has the highest stunting (57.7%) followed by Malawi (47.1%); Niger has the highest wasting (18%), followed by Burkina Faso (15.5%); and Burundi has the highest underweight (28.8%), followed by Niger (36.4%), Chad (28.8%), and Nigeria (28.7%). Ethiopia also has high wasted (8.70%) and underweight (25.2%) among East African countries. In 2010, about 39.4%, 24.9%, and 10.3% of under-five in Africa were stunted, underweight, and wasted, respectively [6]. In 2015, the undernutrition in sub-Saharan African accounts for one-third of the global estimate [7].

According to the Ethiopian Demographic and Health Survey 2016, the Tigray region has 39.3%, 23%, and 11.1% of stunting, underweight, and wasting, respectively. Moreover, 13.4% of under-five were experiencing severe stunting, 3.40% had severe wasting, and 5.20% had severe underweight [8]. Though Ethiopia achieved a Millennium Development Goal 4 (MDG4) of the child mortality reduction, the country still experiences an increment of stunting, wasting, and underweight cases, where the Tigray region had the rates of stunting, wasting, and underweight above the national average [9].

Previous research studies conducted in the Tigray region revealed that 46.9%, 33%, and 11.6% of under-five were stunted, underweight, and wasted [10]. A study conducted on the trend of malnutrition in the Tigray region showed that 33% and 51% of under-five mortality were due to severe acute malnutrition in 2011 and 2015 [11]. Another study done in Adi-Harush and Hitsats refugee camps in the Tigray region indicates 37%, 21.6%, and 11% of children aged 6–59 months were stunted, underweight, and wasted, respectively [12]. A similar study on malnutrition and associated factors of under-five based on hospital data in the west Arsi zone of the Oromia region showed that the magnitude of stunting, underweight, and wasting was about 38.3%, 49.2%, and 25.2%, respectively [13]. On the other hand, in the east Gojjam Zone of the Amhara region, 44.7% of stunting, 15.3% of underweight, and 10.0% of wasting were reported [14].

The determinants of stunting, wasting, and underweight are significantly associated with socioeconomic and demographic characteristics of mothers and children and environmental factors. Maternal education status was found to be strongly inversely associated with under-five stunting, underweight, and wasting [15–22]. Maternal age was also a significant determinant of under-five stunting and underweight [15, 23].

Other determinants of stunting, wasting, and underweight include the place of residency [23], the number of under-five children in households [15, 23, 24], birth order [23, 25], sources of improved drinking water [16, 23], and toilet facility [23]. There is also a strong and positive association between antenatal care service utilization and child stunting/chronic malnutrition [23]. Most studies also found that sex of a child [15, 17, 23, 25], child age [13, 15, 23–25], and household wealth index [15, 17, 24, 26] were found to be significant risk factors of under-five stunting, wasting, and underweight. Further, earlier studies from Ethiopia show that factors associated with increased risk of under-five stunting include baby weight at birth [16], short duration of exclusive breastfeeding [16, 24, 25, 27–29], minimum dietary diversity and meal frequency [19, 30, 31], and diarrhea in the past 2 weeks [17, 24].

Despite the fact that a number of studies have been done on the identification of factors that are associated with under-5 stunting, wasting, and underweight in the Tigray region, none of them uses the nationally representative data for the Tigray region. The progress made in decreasing under-five stunting, wasting, and underweight in the region is still high, and more effort is needed to improve the barriers for further reduction. More research studies are, therefore, required to inform policymakers to implement appropriate intervention programs. To address this gap, we conducted an all-inclusive cross-sectional analysis of the recent 2016 Ethiopian Demographic Health Survey, to assess the risk factors for stunting, wasting, and underweight. Therefore, the main objective of this study was to assess the risk factors associated with stunting, wasting, and underweight of under-five in the Tigray region.

## 2. Materials and Methods

### 2.1. Description of the Study Area

The Tigray National Regional State is located in the northern part of Ethiopia. According to the 2007 Census, the state's population size was 3,136,267 of which 1,594,102 were females. The urban residents of the region were 468, 478 and its rural residents 2,667,789 [32].

### 2.2. Source of Data

The data onto this study were extracted from the Ethiopian Demographic and Health Survey (EDHS) 2016. The Central Statistics Agency (CSA), the Ministry of Health (MOH), and the Ethiopian Public Health Institute together conducted the survey from January 18, 2016–June 27, 2016, where the United States Agency for International Development (USAID) funded the project. The survey implemented a two-stage sample design. In the first stage, 645 enumeration areas were selected with probability related to size. In the second stage, 28 households per cluster of equal probability systematic were selected from the household list. All women of 15–49 years that were either stable inhabitant or visitors, who lived at least one night in the household before the survey, were eligible for the interview. Data were gathered by conducting face-to-face interviews for women that met the eligibility criteria.

### 2.3. Variables of the Study

Determinants of stunting, wasting, and underweight in this study were selected from the available similar studies on the subject; the main predictors explored for under-five nutritional status were grouped into demographic, socioeconomic, and environmental factors related to mothers and households. Then, the nutritional status of a child was calculated based on the three anthropometric indicators: wasting (weight-for-height), stunting (height-for-age), and underweight (weight-for-age). The dependent variables of this study were stunting, wasting, and underweight among children aged 0–59 months.

### 2.4. Statistical Analysis

Data analysis was done using SPSS version 21.0 (2018). The descriptive statistics such as frequencies and proportions were used to summarize the distribution of selected background characteristics of the sample. To estimate the effect of each demographic, socioeconomic, and environmental factors on under-five stunting, wasting, and underweight (odds ratio (OR) with 95% confidence intervals (CI)), logistic regression analysis was fitted. Bivariate analysis based on Pearson chi-square tests was used for testing association with the predictors and outcome variable under-5 stunting, wasting, and underweight. All significant predictor variables (*p* < 0.05) in the bivariate analysis were included in the multivariate logistic regression analysis. The goodness of fit of the fitted models was checked using the Hosmer and Lemeshow test (HLT).

## 3. Results

### 3.1. Descriptive Statistics

More than two-thirds of the children were from mothers aged 15–20 years at first birth (67%). Nearly one child in 10 children was from rural areas (88%) while the remaining 12% of the children were living in urban areas. According to Table 1, children from uneducated mothers have a higher percentage (67.2%) of samples. Similarly, the highest percentage of children (52.5%) belonged to a mother whose husband has no education at all, while only 8% has a secondary or higher education.

The majority (42.0%) of the respondents used unprotected drinking water and 38% of them used protected well while only one-fifth (20%) of respondents had piped sources of drinking water. Similarly, more than half (58.5%) of the respondents had no toilet facility, and 41.5% had toilet facilities. More than half (52.3%) of respondents had two under-five children in the household, more than one-third (36.3%) had one under-five child in households, and only 11.4% of respondents had at least three children less than five years in households in the past five years preceding the survey. More than half (52.8%) belonged to the poor wealth index, while 32% belonged to rich household wealth indices.

About 54% of children belong to mothers that did not attend any antenatal care services during pregnancy, and only one from five (22%) had attended at least four antenatal visits during pregnancy. On the other hand, only 11.40% of mothers have attended postnatal care services. The percentage of age distribution of samples included in the study was almost consistent with all age groups. The vast majority (86%) of children included reported that they had no diarrhea two weeks before the survey. Similarly, about 85% of children born five years before the survey had no anemia. As the vaccination is concerned, 78% of them have received vitamin A in the last 6 months, about 74% have received measles, and about 40% have received tetanus.

Results showed that about 19.4% of the children were first birth, about 44% were second, third, or fourth, and 36% were had birth order at least a fifth. The vast majority (53.5%) of women have been engaged in the agricultural sector while 21% were housewives without a formal job. The proportion of children currently breastfeeding was nearly two-thirds (63%), and only 3% of the under-five included in the study was born through the Cesarean section birth. As high as 89% of children were born at home outside health facilities and more than 97% of births were singleton. Concerning birth weight, about 34% of under-five included in the study had a birth weight less than 2.5 kg, about 46% had 2.50–4 kg, and 20% had more than 4 kg (Table 1).

### 3.2. Bivariate Analysis

**Figure 1:**
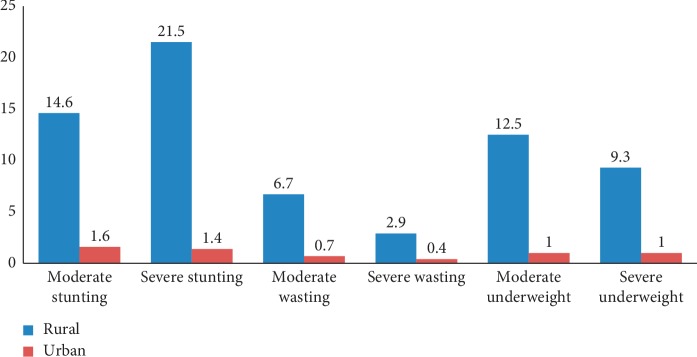
Prevalence of stunting, wasting, and underweight by place of residence in the Tigray region, Ethiopia.

According to Table 2, under-five stunting, wasting, and underweight were higher in the rural areas, and about 92.4% stunted, 89.5% wasted, and 91.4% of underweight were reported among rural children born five years before the survey. As indicated in Figure 1, among rural children, 21.5%, 2.90%, and 9.30% were severely stunted, wasted, and underweight, respectively.

Regarding maternal education, the percentage of stunting, wasting, and underweight among children born of the uneducated mother was relatively higher than that of attending at least a primary education. Among children born to a woman with no education, 71.0%, 71.9%, and 71.6% were stunted, wasted, and underweight, respectively. Similarly, stunting, wasting, and underweight among under-five children differed significantly with the partners' level of education, with those of no education, having a higher proportion of experiencing stunting (58.2%), wasting (52.6%), and underweight (59.9%). Only 2.10%, 1.80%, and 1.90% of children born from a woman whose partner has higher education in the last five years prior to the survey were stunted, wasted, and underweight.

As maternal age was concerned, the highest percentage of under-5 stunting, wasting, and underweight was among children belonging to older age women. About 30%, 23.7%, and 28% of under-five children whose mother aged 35 and older were stunted, wasted, and underweight, respectively, while only 3.50%, 5.30%, and 4.70% of children whose mother is 15–19 years old were stunted, wasted, and underweight, respectively. Similarly, stunting, wasting, and underweight among under-five differed significantly with the sources of drinking water, and those used unprotected well have the highest proportion of stunting (49.4%), wasting (40.3%), and underweight (47.1%), respectively, while only 15.2%, 20.2%, and 15.6% of children belonging to the household using piped source experienced under-five stunting, wasting, and underweight, respectively. On the other hand, 61.8%, 64.9%, and 60.3% of children belonging to a family with no toilet facilities had experienced under-five stunting, wasting, and underweight, respectively (Table 2).

With regard to mother's antenatal care follow-ups and baby postnatal care visits, the result shows that among children born from mothers who were not receiving any antenatal care, 58.4%, 57%, and 61.1% were stunted, wasted, and underweight, respectively, while 92.6%, 96.5%, and 97.7% of stunting, wasting, and underweight were reported among children who had not attended any postnatal checkup. Similarly, looking at birth weight of a child, high proportion of stunting (41.3%), wasting (42.1%), and underweight (48.6%) was associated with lower birth weight (<2.50 kg). About 46%, 55%, and 44% of stunting, wasting, and underweight were reported among children with second, third, or fourth birth order. Also, the percentage of stunting increases with child age between 2 and 4 years and the lowest proportion of stunting was reported among infants.

**Figure 2:**
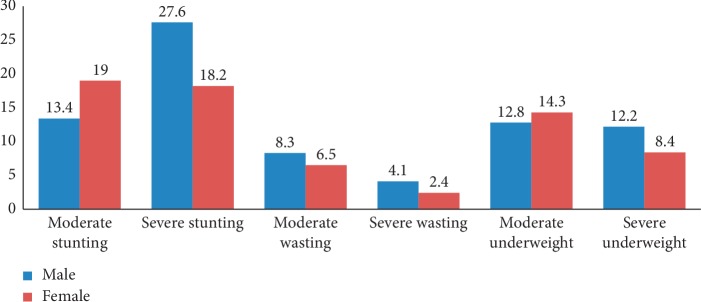
Prevalence of stunting, wasting, and underweight by sex of child in the Tigray region, Ethiopia.

Stunting, wasting, and underweight also vary with the number of under-five children in households, where 49.9%, 55.3%, and 50.6% of under-five from families with two under-five children in families had stunted, wasted, and underweight, respectively. The highest percentage of stunting (49.1%), wasting (48.2%), and underweight (56.8%) was observed among children whose mothers are working in the agricultural sector. Also, high percentages of under-five stunting (36.1%), wasting (28.10%), and underweight (36.6%) were reported among children belonging to households with the poor economic level, unlike minimum percent of stunting (9.30%), wasting (11.4%), and underweight (9.30%) was reported among those children belonging to the richest household. Stunting, wasting, and underweight were also highly variable with the sex of a child in bivariate analysis with male children reporting a higher percentage of stunting (52.5%), wasting (57.9%), and underweight (52.5%). Similarly, 27.6% of male children were severely stunted, 4.10% of male children were severely wasted, and 12.2% of male children were severely underweight (Figure 2).

In the multivariable logistic regression analysis, sources of drinking water, number of under-five children in a family, child birth weight, measles, tetanus, household wealth index, and child age were statistically significant covariates for stunting. The odds of stunting among children from families using protected drinking water were 0.68 (OR = 0.68, 95% CI: (0.50, 0.92)) times lower than those children belonging to families using unprotected drinking water. Only one child (OR = 0.55, 95% CI: (0.23, 0.92)) and two children (OR = 0.47, 95% CI: (0.30, 0.74)) in a family were associated with decreased odds of stunting compared to those children from households with three or more under-five children. Similarly, birth weights >4 kg (OR = 0.36, 95% CI: (0.24, 0.54)) and 2.50–4 kg (OR = 0.60, 95% CI: (0.44, 0.81)) were associated with lower odds of stunting. Household wealth index and age of a child were other variables significantly associated with stunting. Children born in the poorest households (OR = 2.73, 95% CI: (1.26, 5.93)) aged between 12 and 23 months (OR = 1.67, 95% CI: (1.01, 2.77)) and 24 and 34 months (OR = 2.00, 95% CI: (1.29, 3.10)) were highly significantly stunted.

Child weight at birth 2.50–4 kg (OR = 0.62, 95% CI: (0.39, 0.99)) and no diarrhea disease recently (OR = 0.41, 95% CI: (0.25, 0.68)) had lower odds of wasting, while absence of antenatal care visits during pregnancy (OR = 2.20, 95% CI: (1.04, 4.64)) and child age 0–11 months (OR = 3.11, 95% CI: (1.26, 7.67)) and between 12 and 23 months (OR = 2.53, 95% CI: (1.16, 5.55)) had a statistically significantly higher odds of wasting.

Results in Table 3 revealed that 2 under-five children in households (OR = 0.56, 95% CI: (0.35, 0.91)), maternal age at birth less than 20 (OR = 0.66, 95% CI: (0.45, 0.97)), baby weight at birth >4 kg (OR = 0.30, 95% CI: (0.19, 0.49)) and 2.50–4 kg (OR = 0.48, 95% CI: (0.34, 0.67)), no diarrhea disease recently (OR = 0.34, 95% CI: (0.23, 0.52)), the poorest wealth index (OR = 3.21, 95% CI: (1.20, 8.56)), and male sex (OR = 2.04, 95% CI: (1.13, 3.68)) were the factors that have significant impact on underweight (Table 3).

## 4. Discussion

The prevalence of stunting, wasting, and underweight in the region was 39.1%, 10.6%, and 23.9%, respectively. This figure is lower than the previously reported stunting (46.9%), wasting (11.6%), and underweight (33%) in the region [10]. A similar studies conducted in Hidabu Abote District in Oromia region reported a higher prevalence of stunting (47.6%), underweight (30.9%), and wasting (16.7%) [33]. A similar study conducted in rural Ethiopia also reported 41.2% of stunting and 27% of underweight [34], whereas a study conducted in Nigeria reported 47.6% and 25.6% of stunting and underweight, respectively [35]. While a study in the Bure Town of West Gojjam Zone (Amhara region) [17] reported lower prevalence of stunting, wasting, and underweight (24.9%, 11.1%, and 14.30%, respectively), another study in Okrika Town of Nigeria [36] also reported 13.6%, 8.80%, and 10.5% prevalence of stunting, wasting, and underweight, respectively. A similar study in east Gojjam Zone [14] reported lower prevalence of underweight (15.3%) and wasting (10%), but higher stunting (44.7%).

In this study, the prevalence of severe stunting (22.9%) and underweight (10.3%) is markedly higher than the previous studies that reported 7.90% severely stunting and 3.20% severely underweight in Bure Town, west Gojjam Zone [17]. Children born in rural areas five years preceding the survey were more likely severely stunted (21.5%) and severely underweight (9.30%) than their urban counterparts. The possible reason for this might be a lack of healthcare and food insecurity programs in rural areas. Moreover, male children are highly severely stunted (27.3%) and severely underweight (12.2%) than females. This figure is higher than that reported in the study in the Bure Town of west Gojjam [17], 10.7% of severely stunted and 6% of severely underweight.

In the multivariate logistic regression analysis, it was found that being born of a weight less than 2.50 kg was associated with a higher risk of stunting, wasting, and underweight than about average (2.50–4 kg) or larger size at birth (>4 kg). Several studies in the literature reported babies born of smaller size were at higher risk of stunting [15, 37], wasting, and underweight. This might be for the reason that low birth weight is in turn associated with a range of adverse outcomes of first childhood life.

Being born of a mother with a younger age at birth (less than 20 years) was associated with a lower risk of underweight compared to being born of mothers older than 20 years. This finding was in contradiction with the results of those children born of mothers at an earlier age having a higher chance of experiencing under-five underweight [38, 39].

Antenatal care visits during pregnancy were identified as a strong predictor of under-5 wasting in the multivariate analysis after controlling for the effect of other covariates. Results show that children born of mothers that do not attend any antenatal care service at the time of pregnancy have a significantly higher risk of wasting compared to those born of mothers that attended at least four antenatal visits. Previous research studies [17, 26, 40] also revealed that antenatal follow-ups during pregnancy have been significantly associated with a reduced chance of wasting. The reason for this may be access to medical treatments for pregnancy which is helpful for the mother to protect her child from different infections. Another possible explanation for this result might be following antenatal care enables mothers to be aware of the advantages of breastfeeding and other feeding practices of their infants. Moreover, the World Health Organization also recommends that a woman should have at least four antenatal visits by health professionals during pregnancy.

Another significant risk factor attributed to stunting among under-five was wealth indices and source of improved drinking water. Children that were born in poor household were at the risk of stunting and underweight. The possible explanation for this might be mothers from households having the rich or middle wealth status were more likely to provide micronutrients in reached foods and seek medical treatment for their children. Previous literature also reported that the poor wealth index is strongly correlated with under-five stunting [15, 17, 23, 24, 26, 41, 42]. Furthermore, better of households has better access to food and higher cash incomes than poor households, allowing them a quality diet, better access to medical care, and more money to spend on essential nonfood items such as hygiene products. Similarly, unprotected sources of drinking water were strongly associated with a higher risk of stunting. This is consistent with available literature that states that improved sources of drinking water are a strong predictor of child stunting [15, 16, 23].

Child diarrhea was found to be a significant association with wasting and underweight, such that children who had diarrhea in the last six months preceding the survey had an increased risk of wasting and underweight compared to those who did not have diarrhea. This finding is in agreement with studies [17, 24] which show a significant association with diarrhea recently and under-five wasting and underweight. This is due to the fact that diarrhea accelerates the onset of acute malnutrition by reducing food intake and increasing catabolic reactions in the organism.

On the other hand, the high number of under-five children in families was more likely to be associated with under-5 stunting and underweight. Various literature studies indicated that larger under-five children in households were significantly positively associated with stunting and wasting [15, 24]. This may be because the large household size is widely regarded as a risk factor for stunting and underweight particularly for infants and young children due to food insecurity.

Age of a child was independently related to stunting and wasting. Similar previous research studies [23–25] reported that child age had a significant association with stunting and wasting of the children. Being a male child was highly positively associated with underweight. Studies have shown that boys had a significantly worse nutritional status than girls [15, 43, 44]. Mother's occupation, maternal or partner's education level, birth order, place of delivery, birth type, and mode of delivery were not significantly associated with experiencing stunting, wasting, and underweight.

## 5. Conclusions

Children from mothers who were not attending antenatal care during pregnancy were at higher risk of wasting. Thus, treatment of mothers during pregnancy should be given due attention. Children of age 0–11 months and 12–23 months are more at risk of wasting. Thus, efforts should be made to communicate through health and nutrition education, the importance of feeding breast milk exclusively up to 6 months, and thereafter introducing other supplementary nutrient-rich foods.

Interventions that focus on children born with lower weight at birth (<2.5 kg) and children born in rural areas are required for improving the child stunting, wasting, and underweight though and improving healthcare services and food insecurity programs in rural areas. Children who use unprotected drinking water are at high risk of stunting. Thus, efforts should be made to improve access to safe drinking water. Children who have diarrhea two weeks before the date of the survey are significantly vulnerable to wasting and underweight than those who have not. Therefore, efforts should be made in improving environmental sanitation and personal hygiene to prevent exposure to diarrhea.

The authors also recommended further investigation based on trend analysis which had to be conducted to see the trend in under-five stunting, wasting, and underweight to achieve the sustainable development goal targets.

## Figures and Tables

**Table 1:** Background characteristics of respondents in the Tigray Region, Ethiopia (*n* = 1077).

Variables	*N*	Percent
*Sources of drinking water*		
Piped	214	19.90
Protected well	410	38.10
Unprotected well	453	42.10

*Availability of toilet facility*		
No facility/bush/field	630	58.50
With facilities	447	41.50

*Number of under-five children*		
1	391	36.30
2	563	52.30
3+	123	11.40

*Maternal age at birth*		
Less than 20	723	67.10
20 or higher	354	32.90

*Anemia*		
No	918	85.20
Yes	159	14.80

*Mother occupation*		
Not working	223	20.70
Agricultural sector	576	53.50
Other sectors	278	25.80

*Place of delivery*		
Home	961	89.20
Health facilities	116	10.80

*Birth type*		
Single	1049	97.40
Multiple	28	2.60

*Birth weight*		
>4 kg	214	19.90
2.5–4 kg	498	46.20
<2.5 kg	365	33.90

*Had diarrhea recently*		
No	928	86.20
Yes	149	13.80

*Received measles*		
No	281	26.10
Yes	796	73.90

*Vitamin A in last 6 months*		
No	233	21.60
Yes	844	78.40

*Received tetanus*		
No	646	60.0
Yes	431	40.0

*Antenatal follow-up*		
No	576	53.50
1–3	265	24.60
4+	236	21.90

*Postnatal care*		
No	954	88.60
Yes	123	11.40

*Current age of mother*		
15–19	50	4.60
20–24	219	20.30
25–29	277	25.70
30–34	220	20.40
35+	311	28.90

*Husband/partner's education*		
No education	595	55.20
Primary education	395	36.70
Secondary education	49	4.50
Higher education	38	3.50

*Child birth order*		
First birth	209	19.40
2, 3, or 4	477	44.30
Fifth or more	391	36.30

*Place of residence*		
Rural	948	88
Urban	129	12

*Currently breastfeeding*		
No	401	37.20
Yes	676	62.80

*Mode of delivery*		
Normal	1047	97.20
Cesarean section	30	2.80

*Sex of child*		
Male	539	50
Female	538	50

*Household wealth index*		
Poorest	332	30.80
Poorer	237	22
Middle	172	16
Richer	168	15.60
Richest	168	15.60

*Mother education*		
No education	724	67.20
Primary education	297	27.60
Secondary education	40	3.70
Higher education	16	1.50

*Child age*		
0–11 months	202	18.80
12–23 months	200	18.60
24–35 months	2012	19.70
36–47 months	245	22.70
48–59 months	218	20.20

**Table 2:** Bivariate analysis of stunting, wasting, and underweight by background characteristics of the mother and a child.

Background characteristics	Categories	Stunting (%)	Wasting (%)	Underweight (%)
Sources of drinking water	Piped	15.2	20.2	15.6
	Protected well	35.4	39.5	37.4
	Unprotected well	49.4	40.3	47.1

Availability of toilet facility	No facility/bush/field	61.8	64.9	60.3
	With facilities	38.2	35.1	39.7

Number of under-five children	1	34.9	29.8	33.4
	2	49.9	55.3	50.6
	3+	15.2	14.9	16

Mother occupation	Not working	18.3	26.3	17.9
	Agricultural sector	59.1	48.2	56.8
	Other sectors	22.6	25.5	25.3

Birth type	Single	96.4	95.6	95.3
	Multiple	3.60	4.40	4.70

Birth weight	>4 kg	14	18.4	12.1
	2.5–4 kg	44.7	39.5	39.3
	<2.5 kg	41.3	42.1	48.6

Had diarrhea recently	No	84.8	70.2	77.4
	Yes	15.2	29.8	22.6

Antenatal follow-up	No	58.4	57	61.1
	1–3	25.7	29.8	25.7
	4+	15.9	13.2	14.4

Postnatal care	No	96.2	96.5	97.7
	Yes	3.80	3.50	2.30

Current age of mother	15–19	3.60	5.30	4.70
	20–24	19.7	20.2	19.5
	25–29	25.4	30.7	24.1
	30–34	21.4	20.2	23.7
	35+	29.9	23.7	28

Husband/partner's education	No education	58.2	52.6	59.9
	Primary education	37.8	41.2	35.4
	Secondary education	1.90	4.40	2.70
	Higher education	2.10	1.80	1.90

Child birth order	First birth	17.6	14	18.7
	2, 3, or 4	45.6	55.3	43.6
	Fifth or more	36.8	30.7	37.7

Place of residence	Rural	92.4	89.5	91.4
	Urban	7.60	10.5	8.60

Sex of child	Male	52.5	57.9	52.5
	Female	47.5	42.1	47.5

Household wealth index	Poorest	36.1	28.1	36.6
	Poorer	24.5	21.9	23
	Middle	14.5	19.3	16.3
	Richer	15.7	19.3	14.8
	Richest	9.30	11.4	9.30

Mother education	No education	71.3	71.9	71.6
	Primary education	25.4	23.7	25.7
	Secondary education	2.90	2.60	1.90
	Higher education	0.50	1.80	0.80

Child age	0–11 months	10	27.2	15.2
	12–23 months	21.9	28.1	19.8
	24–35 months	24.9	18.4	24.5
	36–47 months	25.2	12.3	21.8
	48–59 months	18.8	14	18.7

**Table 3:** Factors associated with stunting, wasting, and underweight among under-five children in Tigray Regional State, Ethiopia (*n* = 1077).

Variables	Stunting		Wasting		Underweight	
	AOR	95% CI	AOR	95% CI	AOR	95% CI
*Sources of drinking water (unprotected)*						
Piped	0.94	0.57, 1.53	0.22	0.77, 3.25	1.01	0.57, 1.77
Protected well	0.68^*∗*^	0.50, 0.92	0.44	0.75, 1.96	0.91	0.64, 1.28

*Availability of toilet facility (with facilities)*						
No facility/bush/field	0.96	0.71, 1.314	1.36	0.83, 2.22	0.86	0.60, 1.21

*Number of under-five children (3+)*						
1	0.55^*∗*^	0.23, 0.92	0.73	0.33, 1.58	0.61	0.34, 1.07
2	0.47^*∗∗*^	0.30, 0.74	0.82	0.43, 1.58	0.56^*∗*^	0.35, 0.91

*Maternal age at birth (20 or higher)*						
Less than 20	0.99	0.71, 1.41	0.88	0.51, 1.52	0.66^*∗*^	0.45, 0.97

*Anemia (yes)*						
No	0.75	0.51, 1.10	1.42	0.75, 2.68	0.81	0.53, 1.23

*Mother occupation (other sectors)*						
Not working	1.07	0.70, 1.64	1.34	0.73, 2.49	0.79	0.48, 1.27
Agricultural sector	1.18	0.82, 1.68	0.80	0.46, 1.38	0.80	0.53, 1.19

*Birth type (multiple)*						
Single	0.68	0.29, 1.63	0.82	0.26, 2.58	0.55	0.23, 1.33

*Birth weight (<2.5 kg)*						
>4 kg	0.36^*∗∗*^	0.24, 0.54	0.65	0.35, 1.18	0.30^*∗∗*^	0.19, 0.49
2.5–4 kg	0.60^*∗∗*^	0.44, 0.81	0.62^*∗*^	0.39, 0.99	0.48^*∗∗*^	0.34, 0.66

*Had diarrhea recently (yes)*						
No	0.71	0.47, 1.06	0.41^*∗∗*^	0.25, 0.68	0.34^*∗∗*^	0.23, 0.52

*Received measles (yes)*						
No	0.53^*∗*^	0.36, 0.79	0.86	0.47, 1.55	0.78	0.50, 1.21

*Vitamin A in last 6 months (yes)*						
No	0.92	0.64, 1.33	0.77	0.44, 1.34	0.91	0.61, 1.37

*Received tetanus (yes)*						
No	0.71^*∗*^	0.50, 0.99	1.03	0.62, 1.70	0.81	0.55, 1.19

*Antenatal follow-up (4+)*						
No	1.56	0.98, 2.47	2.20^*∗*^	1.04, 4.64	1.49	0.88, 2.53
1–3	1.34	0.87, 2.06	1.89	0.93, 3.87	1.38	0.83, 2.29

*Postnatal care (yes)*						
No	0.95	0.46, 1.94	1.49	0.48, 4.63	2.01	0.79, 5.13

*Current age of mother (35+)*						
15–19	0.56	0.21, 1.50	1.20	0.29, 5.03	1.51	0.51, 4.47
20–24	0.82	0.43, 1.54	0.90	0.34, 2.35	1.29	0.62, 2.65
25–29	0.94	0.56, 1.57	1.10	0.51, 2.39	1.36	0.76, 2.44
30–34	1.03	0.68, 1.55	0.97	0.51, 1.88	1.42	0.90, 2.26

*Husband/partners education (higher education)*						
No education	1.39	0.50, 3.88	1.44	0.21, 9.95	1.13	0.32, 4.03
Primary education	1.41	0.51, 3.89	1.90	0.28, 12.99	1.05	0.30, 3.72
Secondary education	0.63	0.19, 2.13	1.84	0.26, 13.20	0.93	0.23, 3.80

*Child birth order (5* ^*th*^ *or more)*						
First birth	1.18	0.59, 2.36	0.93	0.30, 2.88	0.90	0.41, 1.95
2, 3, or 4	1.29	0.83, 2.01	1.56	0.80, 3.07	0.93	0.57, 1.52

*Place of residence (urban)*						
Rural	1.19	0.54, 2.62	2.32	0.69, 7.81	2.28	0.85, 6.10

*Currently breastfeeding (yes)*						
No	0.95	0.66, 1.37	1.35	0.76, 2.42	0.91	0.60, 1.37

*Mode of delivery (normal)*						
Cesarean section	0.68	0.25, 1.85	0.99	0.17, 5.76	1.30	0.34, 4.93

*Sex of child (female)*						
Male	1.09	0.68, 1.75	1.86	0.82, 4.22	2.04	1.13, 3.68

*Household wealth index (richest)*						
Poorest	2.73^*∗*^	1.26, 5.93	2.03	0.58, 7.08	3.21	1.20, 8.56
Poorer	2.91^*∗∗*^	1.35, 6.25	2.46	0.71, 8.51	2.96	1.12, 7.84
Middle	1.95	0.89, 4.25	3.04	0.88, 10.54	2.47	0.92, 6.64
Richer	2.28^*∗*^	1.09, 4.77	2.61	0.82, 8.33	2.30	0.90, 5.88

*Mother education (higher education)*						
No education	1.79	0.28, 11.44	0.26	0.03, 2.10	0.79	0.12, 5.10
Primary education	2.02	0.32, 12.74	0.20	0.03, 1.61	0.87	0.14, 5.55
Secondary education	2.68	0.42, 17.05	0.18	0.02, 1.77	0.54	0.07, 3.94

*Child age (48–59 months)*						
0–11 months	0.64	0.35, 1.19	3.11^*∗*^	1.26, 7.67	0.79	0.40, 1.56
12–23 months	1.67^*∗*^	1.01, 2.77	2.53^*∗*^	1.16, 5.55	0.99	0.55, 1.76
24–35 months	2.00^*∗∗*^	1.29, 3.10	1.56	0.75, 3.25	1.56	0.95, 2.54
36–47 months	1.47	0.99, 2.20	0.74	0.34, 1.61	1.03	0.64, 1.64

*Marital status (married/living together)*						
Never married/separated/widowed	0.99	0.58, 1.71	1.42	0.58, 3.51	1.77	0.94, 3.33

Hosmer–Lemeshow test	10.39		7.71		12.67	
*p* values	0.24		0.46		0.12	

Reference categories are in parenthesis. ^*∗*^Significant *p* values <0.05; ^*∗∗*^significant *p* values <0.01.

## Data Availability

The data used to support the findings of this study are available from the corresponding author upon request.

## Abbreviations

ANC: Antenatal care
CSA: Central Statistics Agency
EDHS: Ethiopian Demographic and Health Survey
HIV: Human immunodeficiency virus
MDG: Millennium Development Goal
NCHS: National Center for Health Statistics
SD: Standard deviation
WHO: World Health Organization
US: United States
USAID: United States Agency for International Development.

## Additional Points

Since this study is based on secondary data onto EDHS 2016, some important variables may not be included in the study due to the incompleteness of data (high missing values). Moreover, this study uses mothers' recalls of babies, so there may be recalled bias.
